# Patellar instability-induced bone loss in the femoral trochlea is associated with the activation of the JAK1/STAT3 signaling pathway in growing mice

**DOI:** 10.1186/s13018-023-04019-6

**Published:** 2023-07-24

**Authors:** Chen Ma, Wenguan Kou, Zhaoxia Cui, Wenfeng Liu, Changli Liu, Shengjie Wang, Fei Wang

**Affiliations:** 1 Department of Orthopedic Surgery, Third Hospital of Hebei Medical University, Shijiazhuang, 050000 Hebei China; 2 Department of Orthopedic Surgery, Cangzhou People’s Hospital, Cangzhou, 061000 Hebei China; 3 Hebei Medical University, Shijiazhuang, 050000 Hebei China; 4 Department of Orthopedic Surgery, Hengshui People’s Hospital, Hengshui, 053000 Hebei China

**Keywords:** Patellar instability, Trochlear dysplasia, Bone loss, JAK1/STAT3, Mice

## Abstract

**Introduction:**

Patellar instability (PI) at an early age is believed closely correlated with bone loss in the development of the femoral trochlea and can cause trochlear dysplasia. However, the molecular mechanism of PI-induced bone loss has not been established. The Janus kinase (JAK)/signal transducers and activators of transcription (STAT) signaling pathway plays an important role in bone development by regulating the expression of osteoprotegerin (OPG) and receptor activator of nuclear factor kappa B ligand (RANKL). The aim of this study was to explore the association of JAK1/STAT3 signaling to PI-induced subchondral bone loss in the femoral trochlea.

**Methods:**

Four-week-old male C57BL/6 mice were randomly divided into two groups (*n* = 50/group). Mice in the experimental group underwent surgery to induce PI. Distal femurs were collected 2 and 4 weeks after surgery (*n* = 25 knees/each time point, each group). Microcomputed tomography and histological observations were performed to investigate the morphology of the femoral trochlea and changes in bone mass. qPCR, western blot, and immunohistochemistry analyses were performed to evaluate the expression of JAK1, STAT3, RANKL, and OPG in subchondral bone. A t test was performed for the statistical analysis; a *P* value < 0.05 was considered to be statistically significant.

**Results:**

In the experimental group, subchondral bone loss in the femoral trochlea was observed two and four weeks after PI; morphological changes, such as a flatter trochlear groove and an increased sulcus angle, were observed in the femoral trochlea; qPCR, western blot, and immunohistochemistry analyses showed higher expression of JAK1, STAT3, and RANKL and lower expression of OPG (*P* < 0.05).

**Conclusion:**

PI-induced subchondral bone loss in the femoral trochlea and resulted in trochlear dysplasia in growing mice. This bone loss is associated with activation of the JAK1/STAT3 signaling pathway, which weakens the function of osteoblasts and stimulates both formation and function of osteoclasts.

**Supplementary Information:**

The online version contains supplementary material available at 10.1186/s13018-023-04019-6.

## Introduction

The incidence of patellar instability (PI) is 5 to 7 per 100,000 people in the general population, and there is a significant increase in the population group aged 10–17 years, with incidence ranging from 29 to 43 per 100,000 reported by some authors [1, 2]. PI is preceded by osseous and soft-tissue abnormalities, such as patella alta and greater distance between the tibial tubercle and trochlear groove, but it is a consequence of lateral patellar dislocation and rupture of the medial patellofemoral ligament [3, 4]. Moreover, trochlear dysplasia (TD) is closely associated with PI [5, 6], which involves a series of abnormal anatomical morphologies of the distal femur, such as a shallow trochlear groove and a flat slope of the medial or lateral condyle [7, 8]. Fithian et al. [9] reported that approximately 90% of patients with PI presented with TD. The etiology of TD is still not clearly established yet. Previous studies indicated that PI led to secondary TD in growing animal models, and early patellar reduction prevented or compensated for TD [10–12]. Specifically, PI caused abnormal mechanical stress distribution, cartilage degeneration, subchondral bone loss in the femoral trochlea and developmental femoral trochlea [12–14].

The Janus kinase (JAK)/signal transducers and activators of transcription (STAT) system is a critical pathway mediating cellular responses to a variety of cytokines and growth factors [15]. The importance of the JAK/STAT signaling pathway is emerging, especially the JAK1/STAT3 signaling pathway, in bone development, homeostasis, and healing [16]. The JAK1/STAT3 signaling pathway can be initiated by many bone-active cytokines [17, 18]. Studies have focused on how the JAK1/STAT3 signaling pathway stimulates osteoclast formation under inflammatory conditions and thus leads to bone loss [16]. Moreover, recent studies have indicated that the JAK1/STAT3 signaling pathway plays a significant role in osteogenesis. JAK1 inhibition led to a profound decrease in STAT3 phosphorylation and increased bone mass by increasing osteoblast function even under steady-state conditions, but not by directly affecting osteoclasts. In addition, the expression of OPG and receptor activator of RANKL was critical in the regulation of bone mass [19] and was regulated by the JAK1/STAT3 signaling pathway [20]. To date, there have been no reports on the effects of the JAK1/STAT3 signaling pathway on subchondral bone loss in the femoral trochlea.

Therefore, we investigated the expression pattern of JAK1/STAT3 pathway components in subchondral bone after PI. We hypothesized that the bone loss in the femoral trochlea might be associated with activation of the JAK1/STAT3 signaling pathway, leading to a change in trochlear morphology.

## Materials and methods

### Study design

This study was approved by the Ethics Committee of the Third Hospital of Hebei Medical University. Four-week-old male C57BL/6 mice were obtained from the Laboratory Animal Center of Hebei Medical University. The growth phase corresponds to adolescence in human beings, which is known to be an age range in which the patient population shows the highest incidence of PI [1, 21]. In this study, mice (*n* = 100) were randomly divided into two groups: the control group (*n* = 50) and the experimental group (*n* = 50). Mice were euthanized via an overdose of anesthesia 2 and 4 weeks after surgery (*n* = 25 at each timepoint for each group). In each group, right distal femur tissues were collected (*n* = 25 knees/each time point, five knees for micro-CT analysis, five knees for histochemical staining, five knees for immunohistochemistry assay, five knees for immunohistochemistry qPCR analysis, and five knees for western blotting). According to the animal welfare principles, the sample size cannot be excessive. Therefore, we used 5 knees for each of these analyses.

### Surgical technique

In this study, all mice were anesthetized with pentobarbital sodium (50 mg/kg, intraperitoneal injection), and then, a spot was shaved and sterilized carefully. In the control group, a simple incision and suture operation was performed on the right knees of the mice underwent. In the experimental group, surgery to induce PI was performed on the right knees of the mice. A 1-cm incision was made along the midline of the skin around the knee. After dissecting the skin and subcutaneous tissue, the joint capsule and patellofemoral joint were observed. The surgical technique for inducing PI was similar to that described in our previous studies [12, 14, 22]. Specifically, a 0.5-cm longitudinal incision was made between the medial margin of the patella and medial capsule, the retinaculum. Thus, PI was achieved during surgery. Then, the incision was closed with a 5-0 absorbable suture. Mice received acetaminophen (30 mg/kg daily) for five days to control postsurgical pain. All mice underwent treadmill training for 20 min twice every day after three days of surgery to prevent disuse osteoporosis and joint stiffness.

### Microcomputed tomography (micro-CT) analysis

**Fig. 1:**
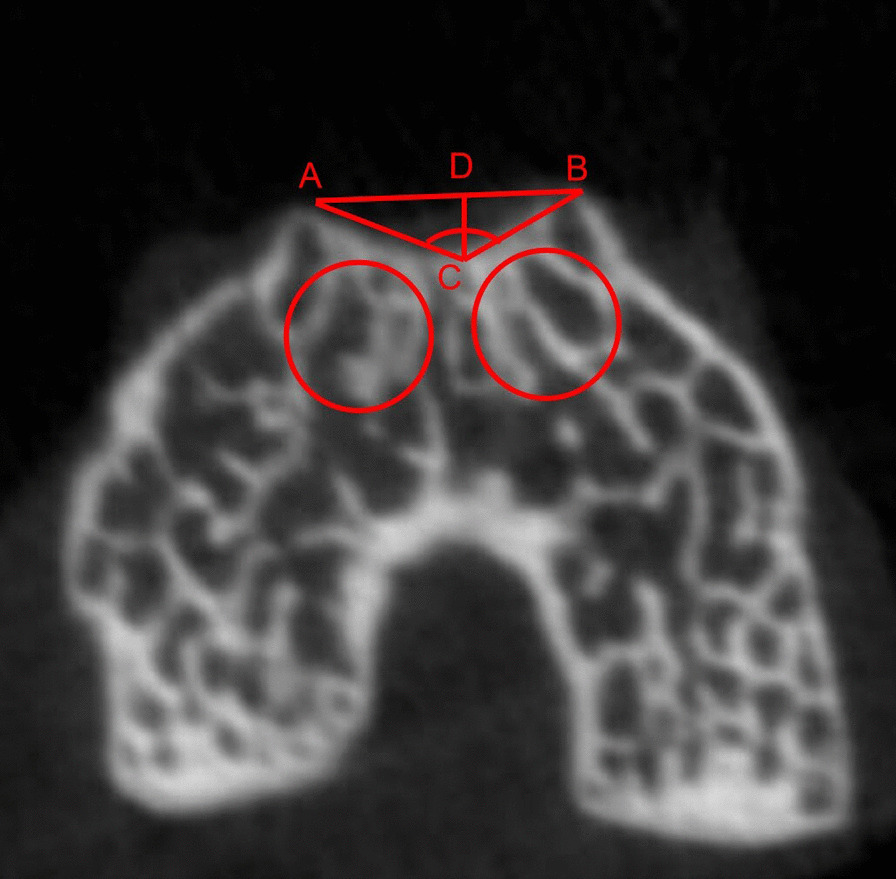
An axial view of the femoral trochlea. ACB, sulcus angle; CD, trochlear depth

Bone tissues of all right distal femurs were carefully harvested 2 and 4 weeks after surgery. Then, they were scanned via micro-CT (SkyScan Model 1076, SkyScan, Belgium). The parameters of micro-CT were set at a 10-µm voxel size, 50 kV, and 800 µA. TD was confirmed with micro-CT images on the basis of the diagnostic criteria defined by Dejour et al. [23]. The depth and sulcus angle of the trochlea were also measured (Fig. 1). The sulcus angle is the angle constituted between the lowest point of the femoral trochlea and the highest point of medial and lateral condyles. The trochlear depth is defined as the distance from the connecting line of the medial and lateral femoral condyles to the deepest part of the trochlear groove [24, 25]. The region of interest (ROI) is indicted by two red cylinders approximately 1 mm in diameter, and the height of the region was approximately 1 mm (starting from 0.5 mm distal to the growth plate) and was located transversely under the medial and lateral facets of the trochlea to analyze microstructural parameters, and the following representative parameters were assessed to determine the growth of subchondral bone, as described in previous studies [26, 27]: bone volume density (BV/TV, %), average trabecular number (Tb. N, 1/µm), average trabecular thickness (Tb. Th, µm), and bone mineral density (BMD).

### Histological staining

Samples were collected at each time point. Then, they were fixed with 4% polyformaldehyde, decalcified in 10% EDTA until completely demineralized, and embedded in paraffin. Sections (5 µm) were cut along the femoral axis to obtain transverse images of the trochlear sulcus. Then, the slices were stained with hematoxylin and eosin (HE) to evaluate subchondral bone damage, and TRAP staining was performed to identify osteoclasts. The slices were observed with a microscope, and representative sections of these slices were photographed with a camera. The results were analyzed with ImageJ Software (ImageJ, USA).

### qPCR

Subchondral bone was carefully excised from the distal femur with a small sharp knife. RNA was extracted from subchondral bone using TRIzol reagent (Servicebio, China). A RevertAid™ first-strand cDNA synthesis kit (K1622, Thermo, USA) was used to translate mRNA into cDNA. The target genes were JAK1, STAT3, RANKL, and OPG, and a sequence detection system was used for gene analysis. The expression of target mRNA was standardized relative to the GAPDH gene and calculated with the formula 2^−ΔΔCt^ (cycle threshold method). The primers used are listed in Table 1. Each experiment was performed three times, and the average values were obtained.

**Table 1:** Primers for amplification of target genes and GAPDH

	Forward primer sequence	Reverse primer sequence
JAK1	CCTGGAGTGGAGGTGACTTTCTA	GTTCGTTGTCATTGGTTCCGT
STAT3	CTGGTGTGAACTACTCAGGGTGT	GGGCTTTGTGCTTAGGATGG
RANKL	CCATCGGGTTCCCATAAAGTCA	CAGTTTTTCGTGCTCCCTCCTT
OPG	CCCTTGCCCTGACCACTCTTAT	AACTGTGTTTCGCTCTGGGGTT
GAPDH	CCTCGTCCCGTAGACAAAATG	TGAGGTCAATGAAGGGGTCGT

### Western blotting

Samples were frozen in liquid nitrogen and then pulverized. Subsequently, they were lysed with lysis buffer supplemented with a protease inhibitor cocktail to prepare protein extracts. Total proteins were extracted using a total protein sample kit (Servicebio, China), and the protein concentration was measured by the bicinchoninic acid method according to a kit manufacturer’s protocols. Next, total proteins were separated via sodium dodecyl sulfate‒polyacrylamide gel electrophoresis (SDS‒PAGE) and then transferred to polyvinylidene fluoride membranes. The membranes were incubated with primary antibodies against JAK1 (AF7765, Affinity, China), STAT3 (GB11176, Servicebio, China), OPG (GB113749, Servicebio, China), and RANKL (23,408–1-AP, Proteintech, China) at a dilution of 1:200 and washed with TBST (5 min per wash, three times). Next, the membranes were incubated with the secondary antibodies at room temperature for one hour. Finally, the target protein bands were analyzed using the Bio–Rad ChemiDoc XRS system (Bio-Rad).

### Immunohistochemistry

The slices were deparaffinized in xylene, rehydrated with ethanol, and washed with phosphate-buffered saline (5 min per wash, three times) at room temperature. Hydrogen peroxide (3%) was used to treat the slices for 10 min to block the activity of endogenous peroxidase. Antigen retrieval was performed by microwave treatment for 10 min in 10 mm of sodium citrate (pH 6.0). Then, slices were incubated overnight at 4 °C with anti-JAK1 (Proteintech, Wuhan, China) and anti-STAT3 antibodies (Proteintech, Wuhan, China) at a dilution of 1:50. Negative controls did not include a primary antibody. Then, photographs of five random sections of each slice were taken with the same background. The percentage of JAK1 and STAT3 positive cells to total cells was calculated. ImageJ software was used as the image analysis tool.

### Statistical analysis

According to a G-Power software estimation, a sample size ≥ 3 is statistically significant. All substantial data are expressed as the means ± standard deviations (SDs). Student’s t test was performed for comparisons between two groups; a *P* value < 0.05 was considered to be statistically significant. SPSS 13.0 software was used for the statistical analysis.

## Results

### Gross morphology and micro-CT assessment

**Fig. 2:**
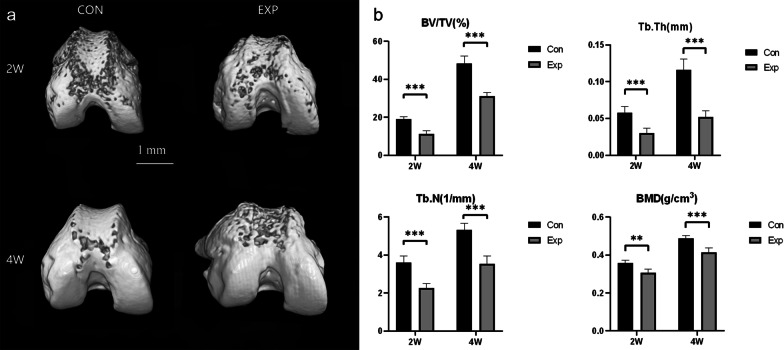
The micro-CT results. **a.** Three-dimensional reconstruction of the distal femur by micro-CT. In the EXP group, the trochlear groove was flat, and bone loss was observed and increased over time. (CON, control; EXP, experimental) **b.** Comparison of the architectural parameters of subchondral bone between the two groups 2 and 4 weeks after surgery as determined via micro-CT. The bone mass increased in the two groups over time. However, compared with the CON group, subchondral bone loss was significant in the EXP group at both 2 and 4 weeks. *P < 0.05, **P < 0.01, ***P < 0.001 (*BV/TV* Bone volume to total volume, *TB* Th: trabecular thickness, TB. N: trabecular number, *BMD* Bone mineral density

We successfully established an experimental model of PI, which further induced TD with a flat trochlear groove and a large sulcus angle (Tables 2, Fig. 2). The sulcus angle and trochlear depth between the two groups showed significant differences two weeks and four weeks after surgery (*P* < 0.05).

**Table 2:** The measurements of sulcus angle and trochlear depth in the two groups (the mean ± standard deviation)

		Control group	Experimental group	*P* value
Sulcus angle	2 weeks	120.76° ± 1.11°	129.20° ± 2.02°	< 0.001
	4 weeks	127.73° ± 1.13°	140.18° ± 2.93°	< 0.001
Trochlear depth	2 weeks	0.21 mm ± 0.012 mm	0.14 mm ± 0.009 mm	< 0.001
	4 weeks	0.28 mm ± 0.013 mm	0.19 mm ± 0.013 mm	< 0.001

### Subchondral bone loss detected by micro-CT and HE staining

**Fig. 3:**
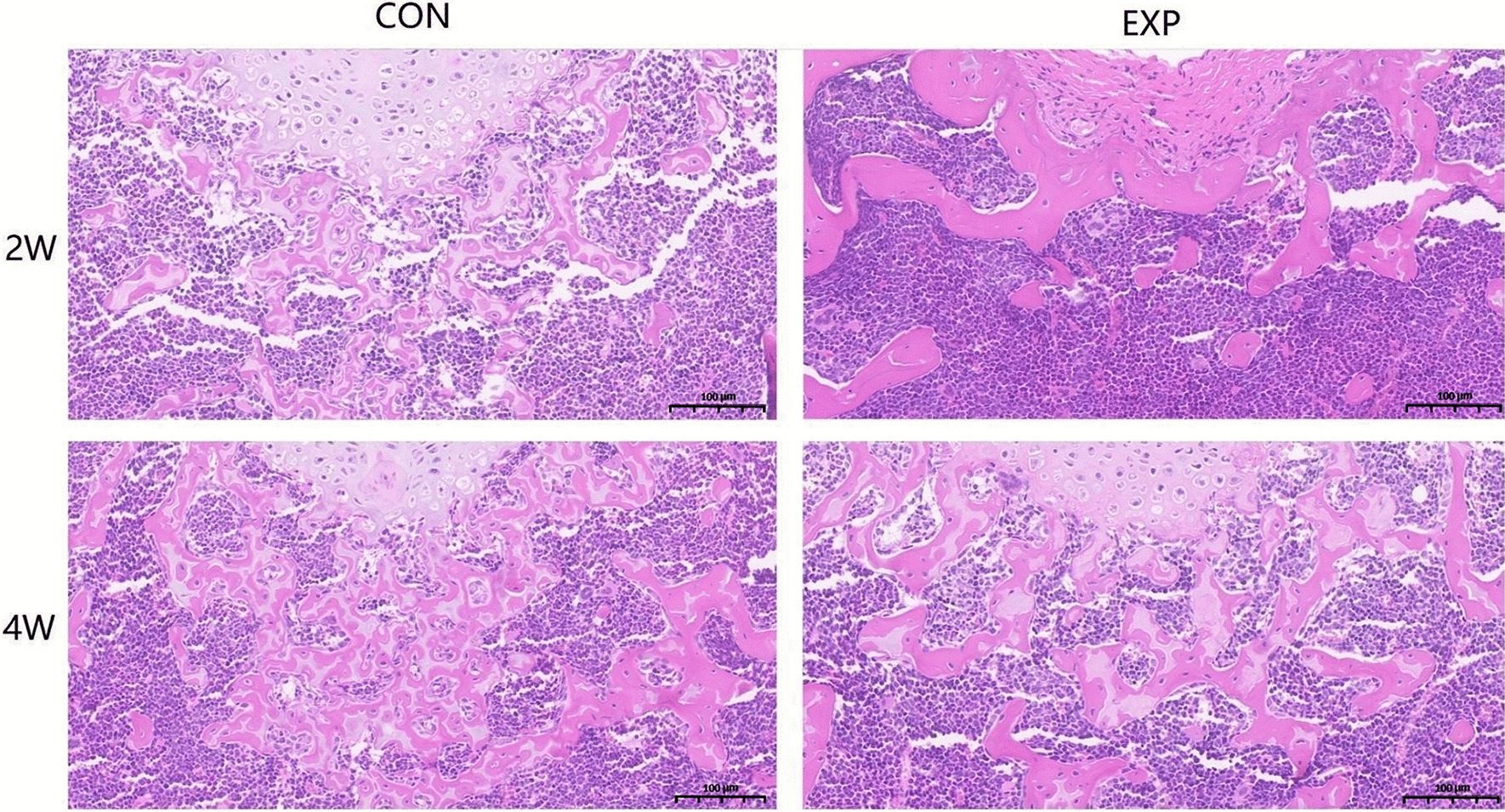
HE staining. Images of HE staining showing trabecular bone loss in the experimental group compared to the time-matched control group. *n* = 5 for each group

Subchondral bone loss was observed in the experimental group by micro-CT, and the bone loss was found to deteriorate over time (Fig. 2A). The micro-CT assay suggested that the BV/TV, Tb.N, Tb.Th and BMD decreased in the experimental group compared to the time-matched control group (Fig. 2B). Images of HE stained section also confirmed the loss of trabecular bone mass (Fig. 3). Histomorphometric results of the micro-CT analysis revealed that BV/TV and TB. Th, TB. N and BMD increased over time in both groups. However, when compared with the time-matched control group, all of the parameters in the experimental group were found to be significantly lower (*P* < 0.05).

### TRAP-positive osteoclast number in subchondral bone increased

**Fig. 4:**
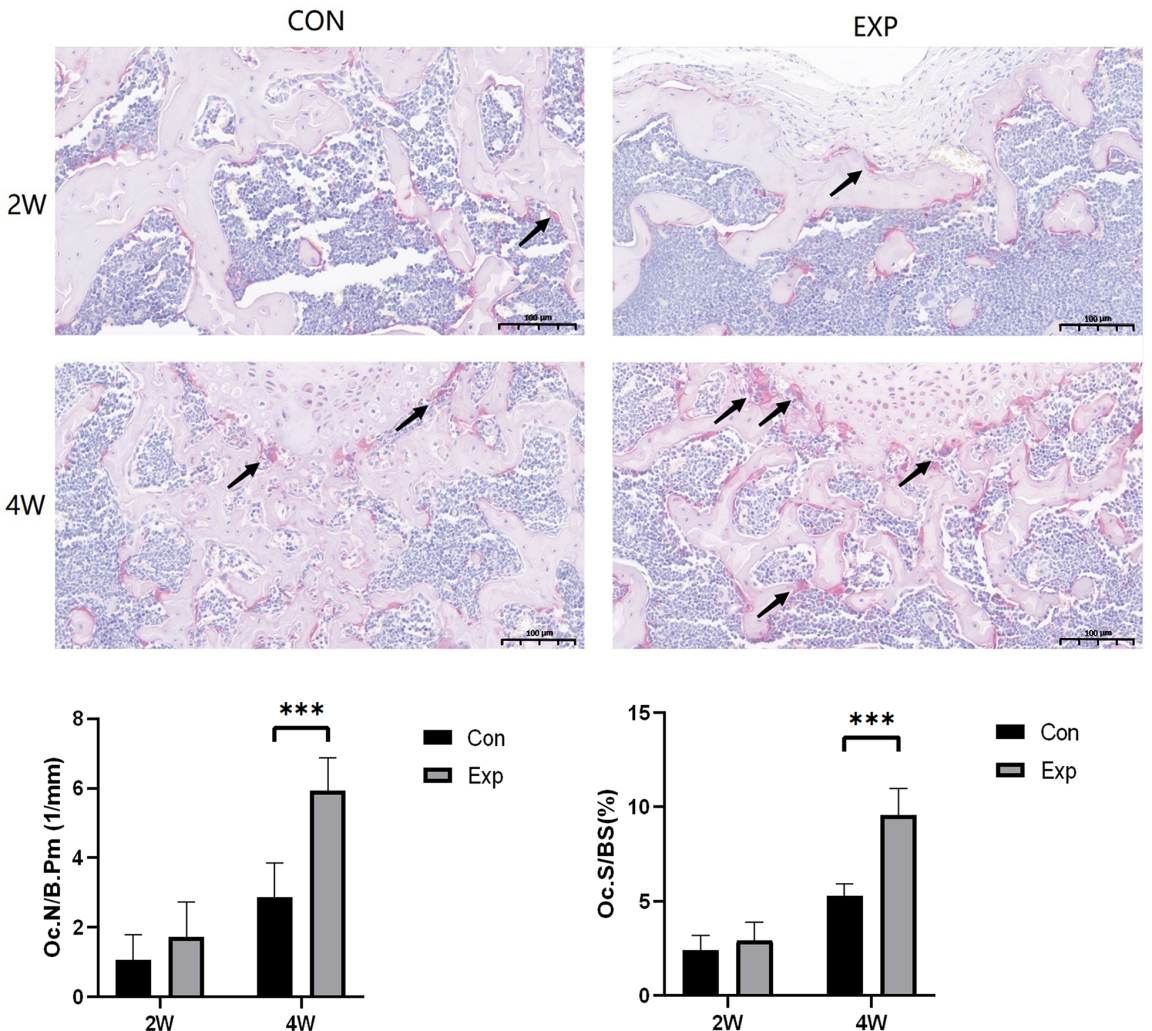
TRAP staining. TRAP staining revealed osteoclasts in the trochlear subchondral bone in the CON and EXP groups at 2 and 4 weeks. Black arrows point to TRAP-positive osteoclasts. Osteoclast number per bone perimeter (Oc. N/B. Pm) and osteoclast surface of bone surface (Oc. S/BS) in the subchondral bone in the CON and EXP groups were significantly different at four weeks. *n* = 5 for each group. *P < 0.05, **P < 0.01, ***P < 0.001

The TRAP staining results showed that the number of TRAP-positive osteoclasts in subchondral bone of the experimental group was low at two weeks, similar to the control group. However, the number of TRAP-positive osteoclasts increased at four weeks in the two groups, especially in the experimental group. Similarly, the morphometric analysis results indicated significant differences in bone resorption parameters, osteoclast number per bone perimeter, and osteoclast surface per bone surface through four weeks (Fig. 4).

### Increased expression of JAK1, STAT3, and RANKL and decreased expression of OPG in subchondral bone

**Fig. 5:**
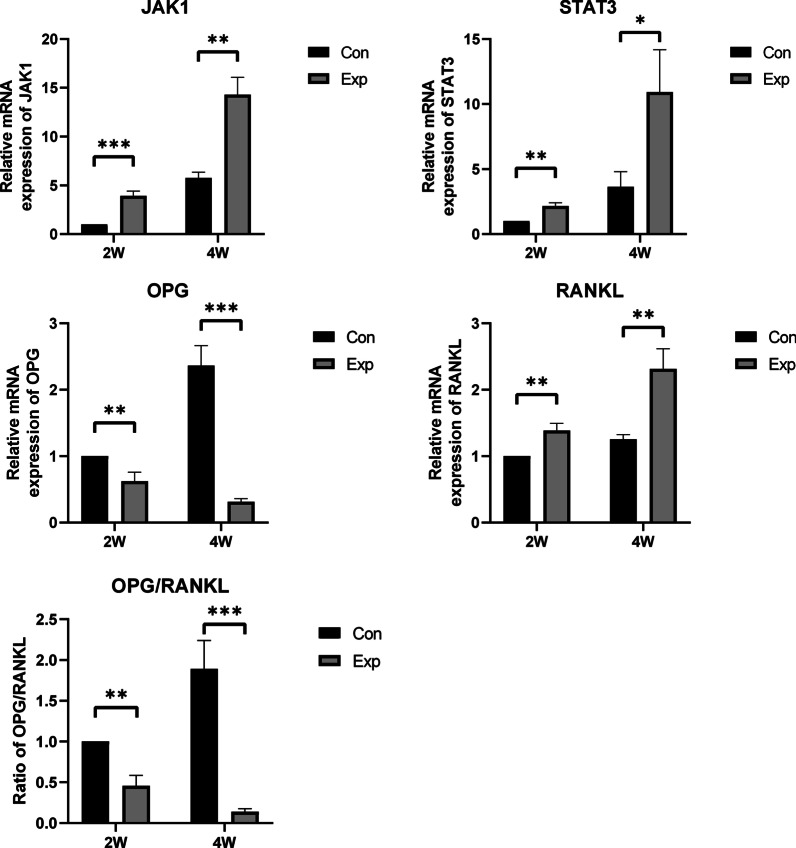
qPCR analysis of relative JAK1, STAT3, OPG, and RANKL mRNA expression in the two groups 2 and 4 weeks after surgery. The OPG/RANKL ratio was significantly decreased in the EXP group. *n* = 5 for each group. *P < 0.05, **P < 0.01, ***P < 0.001

**Fig. 6:**
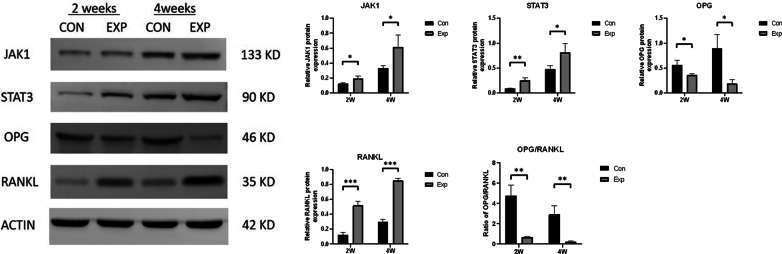
The protein expression of JAK1, STAT3, OPG, and RANKL in subchondral bone of the CON and EXP groups 2 and 4 weeks after surgery. The OPG/RANKL ratio was significantly decreased in the EXP group. *n* = 5 for each group. *P < 0.05, **P < 0.01, ***P < 0.001 (the original blots are presented in Additional file 1: Fig. S1)

**Fig. 7:**
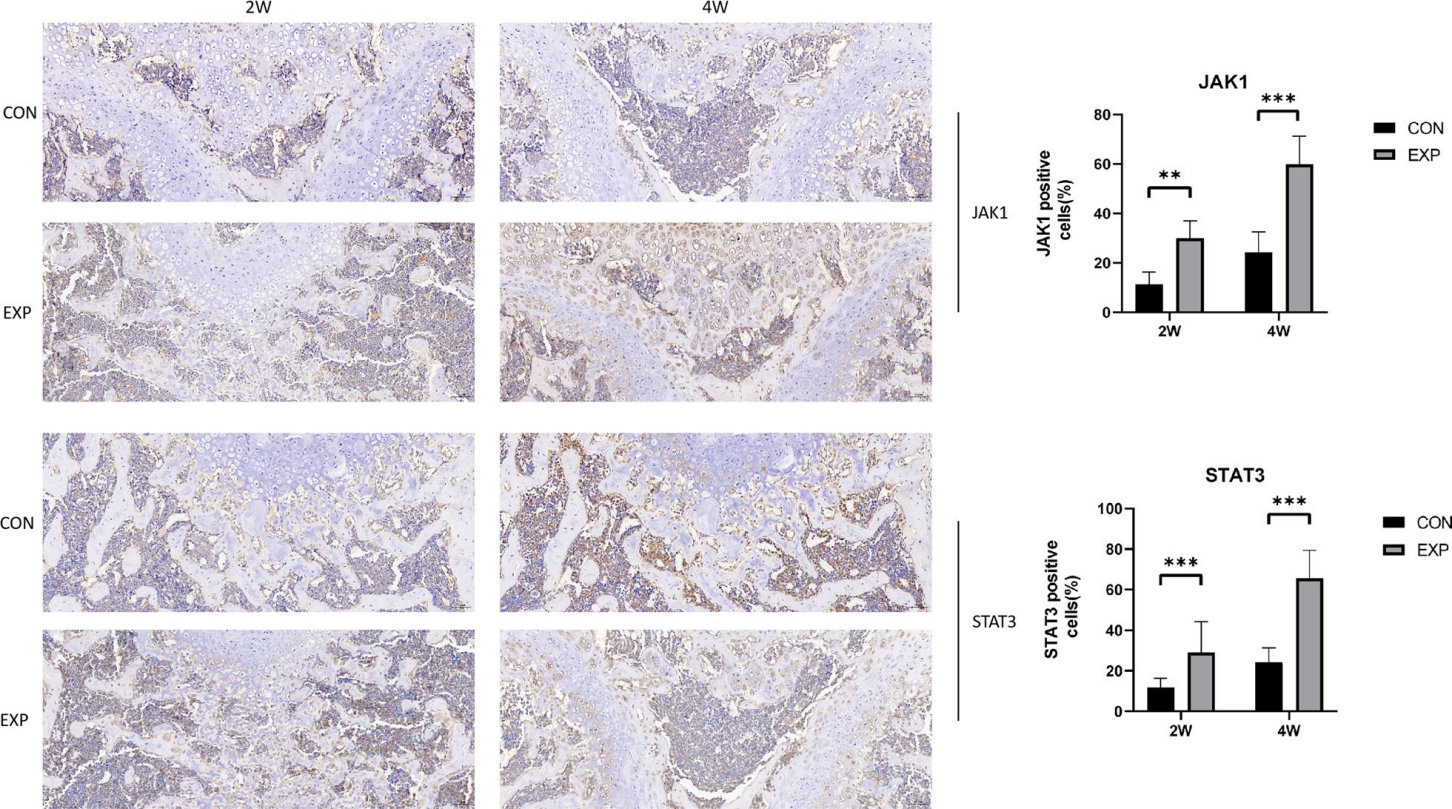
Immunohistochemical analysis of JAK1 and STAT3 2 and 4 weeks after surgery. Black arrows indicate positive expression on subchondral bone. Brown indicates positive staining. Staining intensity analysis of the two groups showed significant differences in JAK1 and STAT3 expression 2 and 4 weeks after surgery. *n* = 5 for each group. *P < 0.05, **P < 0.01, ***P < 0.001

qPCR analysis of the mRNA levels revealed that the expression of JAK1, STAT3, and RANKL in the experimental group was increased, and the expression of OPG was decreased compared with the time-matched control group, resulting in a significant decrease in the OPG/RANKL ratio (*P* < 0.05) (Fig. 5). Additionally, the western blot analysis showed similar expression patterns. (*P* < 0.05) (Fig. 6). The immunohistochemical analysis revealed marked differences in JAK1 and STAT3 levels between the two groups 2 and 4 weeks after surgery (*P* < 0.05) (Fig. 7).

## Discussion

In this study, PI-induced subchondral bone loss in the femoral trochlea, which showed a flatter trochlear groove and larger sulcus angle, and ultimately resulted in TD. Moreover, the most important finding revealed the expression of JAK1/STAT3 was significantly elevated in PI, which indicated that bone loss might have been associated with the activation of the JAK1/STAT3 signaling pathway. To the best of our knowledge, this is the first report considering the importance of JAK1/STAT3 signaling in PI-induced subchondral bone loss and the development of the femoral trochlea in the growing period.

The growth and development of bone is a complicated process controlled by genetic, biochemical, and mechanical factors [28]. Among them, mechanical stress plays a crucial role in promoting osteogenesis and maintaining bone homeostasis [29]. Moreover, the bone tissue in the growing period is more sensitive to mechanical stress [30]. Regarding developmental TD, mechanical stress may play a critical role, since PI causes insufficient sliding stress in the patellofemoral joint and leads to subchondral bone loss [14]. In the present study, with the development of the femoral trochlea, subchondral bone loss was increasingly evident in the experimental group, and morphological malformation was increasingly obvious, which was consistent with previous studies [25, 32]. Also, the changes of subchondral bone to altered mechanical stress follow the general principles of ‘‘bone functional adaptation” [31, 32]. Taken together, we found that persistent abnormal stress leads to bone loss in the femoral trochlea and affects the development of bone, resulting in TD.

The JAK1/STAT3 signaling pathway is crucial in intracellular signal transduction for various cytokines [33, 34] and can affect bone homeostasis [35]. The participation of the JAK1/STAT3 signaling pathway in mechanotransduction has been shown in some cell types, such as chondrocytes, cardiomyocytes, and fibroblasts [36–38], and may also exist in osteoblasts; therefore, further study is needed. The results of micro-CT and HE staining showed subchondral bone loss in the experimental group, accompanied by a high expression of JAK1 and STAT3 in the early stage of TD. However, according to the TRAP staining results, there was little difference in the number of osteoclasts between the control and experimental groups two weeks after surgery. These results indicated that the activation of the JAK1/STAT3 signaling pathway led to a reduction in bone mass, possibly by weakening the function of osteoblasts. Similarly, a previous study revealed that JAK inhibition directly supported osteoblast bone-forming function, but did not inhibit the differentiation or function of osteoclasts [20]. Subchondral bone loss was more severe four weeks after surgery. The results of TRAP staining showed the number of osteoclasts and Oc. S/BS significantly increased in the experimental group, indicating that the formation or function of osteoclasts has also been stimulated. We hypothesized that TD aggravates PI, which further activates the JAK1/STAT3 signaling pathway. The activation of JAK1/STAT3 signaling pathway in PI is associated with shifting the balance between osteogenesis and osteoclastogenesis toward abnormal bone remodeling. These effects may be achieved by decreased osteoblastic bone formation and increased osteoclastic bone resorption.

OPG/RANKL/RANK signaling has been considered the most important pathway in osteogenesis. RANKL stimulates osteoclast formation and maturation; OPG, mainly produced in osteoblasts, can inhibit the effect of RANKL. The balance of OPG and RANKL plays a vital role in maintaining bone metabolism, and the OPG/RANKL ratio reflects the trend in osteogenesis [39]. In our study, the OPG/RANKL ratio was decreased in the experimental group, with high expression of JAK1 and STAT3, indicating that the JAK1/STAT3 signaling pathway may have regulated osteogenesis in this way. Besides, the activation of the JAK1/STAT3 signaling pathway has been previously shown to induce RANKL expression in osteoclast precursors, promoting their differentiation [16, 40], which re-confirmed our findings.

Considering these studies, we speculated that the activation of the JAK1/STAT3 signaling pathway plays an essential role in bone loss in the development of the femoral trochlea. Two weeks after surgery, subchondral bone loss might have been caused mainly by the abrogation of osteogenesis. Four weeks after surgery, subchondral bone loss might have been due to the stimulated osteoclast and diminished osteoblast activity. In either case, the JAK1/STAT3 signaling pathway was involved in regulating bone mass, caused morphological changes in the femoral trochlea, and ultimately resulted in TD. This study provides some insights into the mechanism of bone loss in developmental TD and may lay a theoretical basis for novel therapeutic options.

Recent studies suggested that JAK inhibition could significantly increase bone mass and improve bone microstructure in multiple conditions [16, 41]. Farr JN et al. found that JAK inhibitors could prevent age-related bone loss in mice via targeting cellular senescence [42]. Another study pointed out that JAK inhibition not only increases bone mass in steady-state conditions but also ameliorates pathological bone loss (including post-menopausal osteoporosis or inflammation) by stimulating osteoblast function [20]. As our study has found that JAK1/STAT3 signaling pathway is essential for the growth and development of femoral trochlea in PI, the JAK inhibition may hold promise in reversing bone loss and attenuating TD. However, few studies have addressed the JAK inhibition in growing period due to the importance of JAK signaling pathway in homeostasis and developmental processes such as hematopoiesis, immune development, stem cell maintenance, organ growth, and mammary gland development [43–45]. Besides, systematic application of JAK inhibitor would inevitably affect overall bone formation, especially endochondral bone formation. Topical application of subchondral bone-specific or subchondral bone-targeted inhibition may be an ideal promising therapy. Thus, additional studies would be necessary to examine whether and how JAK inhibition is applicable in PI-induced bone loss and TD.

The current study has some limitations that will be topics of future studies. First, the study using an animal model of disease cannot completely recapitulate human disease in the clinic, verification of these findings in clinical specimens may be necessary in further investigations. Second, as our study showed that bone loss in a dose-dependent manner, our analysis only assessed the early phase of trochlear dysplasia, four weeks after surgery, and a longer-term effect may be evaluated. Finally, our results revealed the potential mechanism of PI-induced bone loss in TD, and additional research is needed to investigate whether JAK inhibition could be served as a novel potential treatment.

## Conclusion

Our study was the first, to our knowledge, to find that PI-induced subchondral bone loss in the femoral trochlea is associated with the activation of the JAK1/STAT3 signaling pathway in growing mice. The subchondral bone loss might have been due to the stimulated osteoclast and diminished osteoblast activity. Our study provided novel insights into the mechanism of bone loss in developmental TD and may lay a theoretical basis for future therapeutic options.

## Supplementary Information

**Additional file 1. Fig. S1a:** The original blots/gels of JAK1, The figure in the munuscript was cropped from the first one. The red circle on the left corresponded to the red circle on the right. **Fig. S1b:** The original blots/gels of STAT3, The figure in the munuscript was cropped from the first one. The red circle on the left corresponded to the red circle on the right. **Fig. S1c:** The original blots/gels of OPG, The figure in the munuscript was cropped from the first one. The red circle on the left corresponded to the red circle on the right. **Fig. S1d:** The original blots/gels of RANKL, The figure in the munuscript was cropped from the first one. The red circle on the left corresponded to the red circle on the right. **Fig. S1e:** The original blots/gels of ACTIN, The figure in the munuscript was cropped from the first one. The red circle on the left corresponded to the red circle on the right.

## Data Availability

The datasets used and/or analyzed during the current study are available from the corresponding author on reasonable request.

## Abbreviations

PI: Patellar instability
TD: Trochlear dysplasia
JAK: Janus kinase
STAT: Signal transducers and activators of transcription
OPG: Osteoprotegerin
RANKL: Receptor activator of nuclear factor kappa B ligand
micro-CT: Microcomputed tomography
ROI: Region of interest
BV/TV: Bone volume density
Tb. N: Average trabecular number
Tb. Th: Average trabecular thickness
BMD: Bone mineral density
HE: Hematoxylin–eosin staining
CON: Control
EXP: Experimental
Oc. N/B. Pm: Osteoclast number per bone perimeter
Oc. S/BS: Osteoclast surface per bone surface
