# Supplementary material for: Patellar instability-induced bone loss in the femoral trochlea is associated with the activation of the JAK1/STAT3 signaling pathway in growing mice
Source: J Orthop Surg Res. 2023 Jul 24;18:526. doi: 10.1186/s13018-023-04019-6 (PMC10364393; doi:10.1186/s13018-023-04019-6)
Supplement: Supplementary file 1 — Additional file 1. Fig. S1a: The original blots/gels of JAK1, The figure in the munuscript was cropped from the first one. The red circle on the left corresponded to the red circle on the right. Fig. S1b: The original blots/gels of STAT3, The figure in the munuscript was cropped from the first one. The red circle on the left corresponded to the red circle on the right. Fig. S1c: The original blots/gels of OPG, The figure in the munuscript was cropped from the first one. The red circle on the left corresponded to the red circle on the right. Fig. S1d: The original blots/gels of RANKL, The figure in the munuscript was cropped from the first one. The red circle on the left corresponded to the red circle on the right. Fig. S1e: The original blots/gels of ACTIN, The figure in the munuscript was cropped from the first one. The red circle on the left corresponded to the red circle on the right. [file 13018_2023_4019_MOESM1_ESM.pdf]

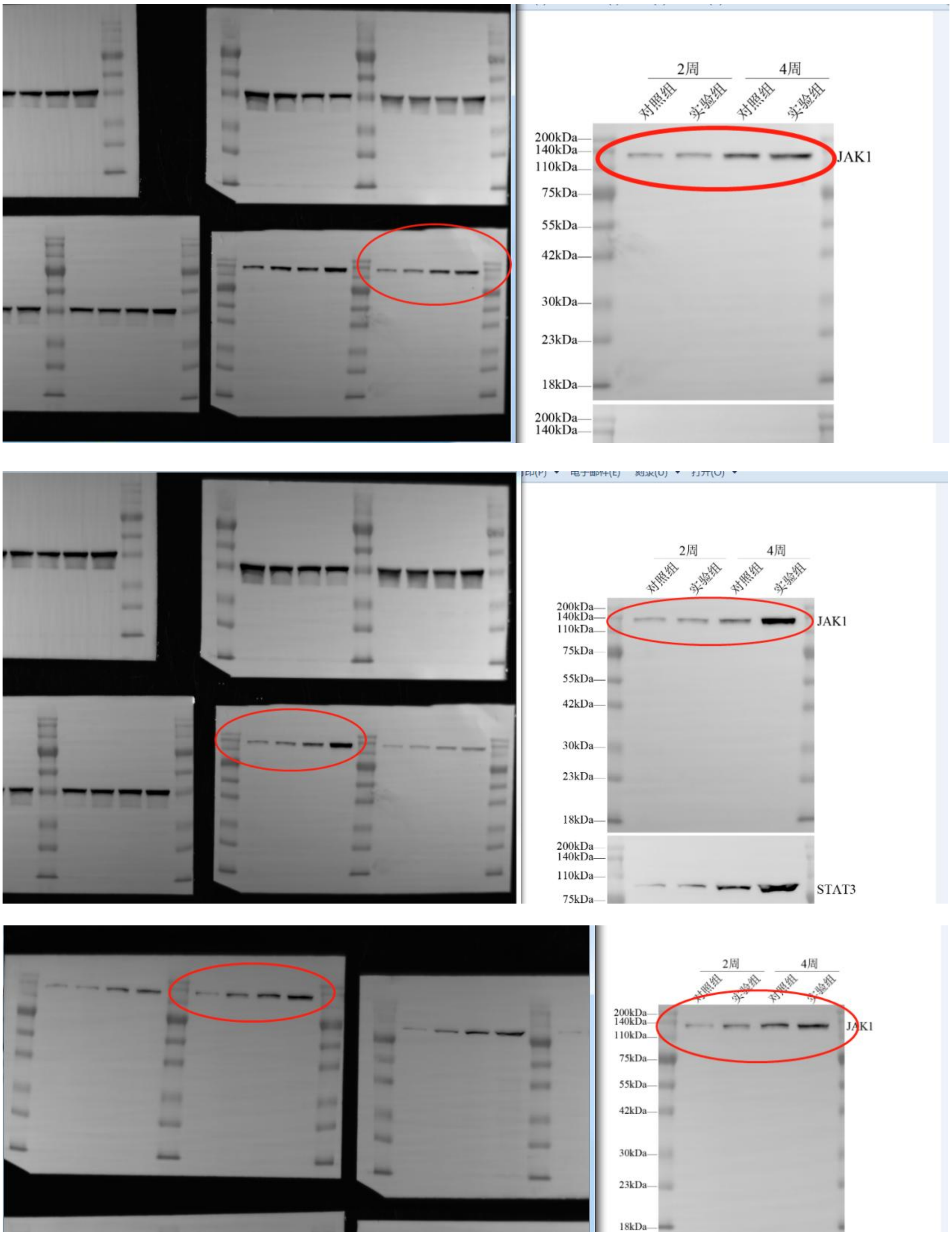

Fig. S1a: The original blots/gels of JAK1, The figure in the munuscript was cropped from the first one. The red circle on the left corresponded to the red circle on the right.

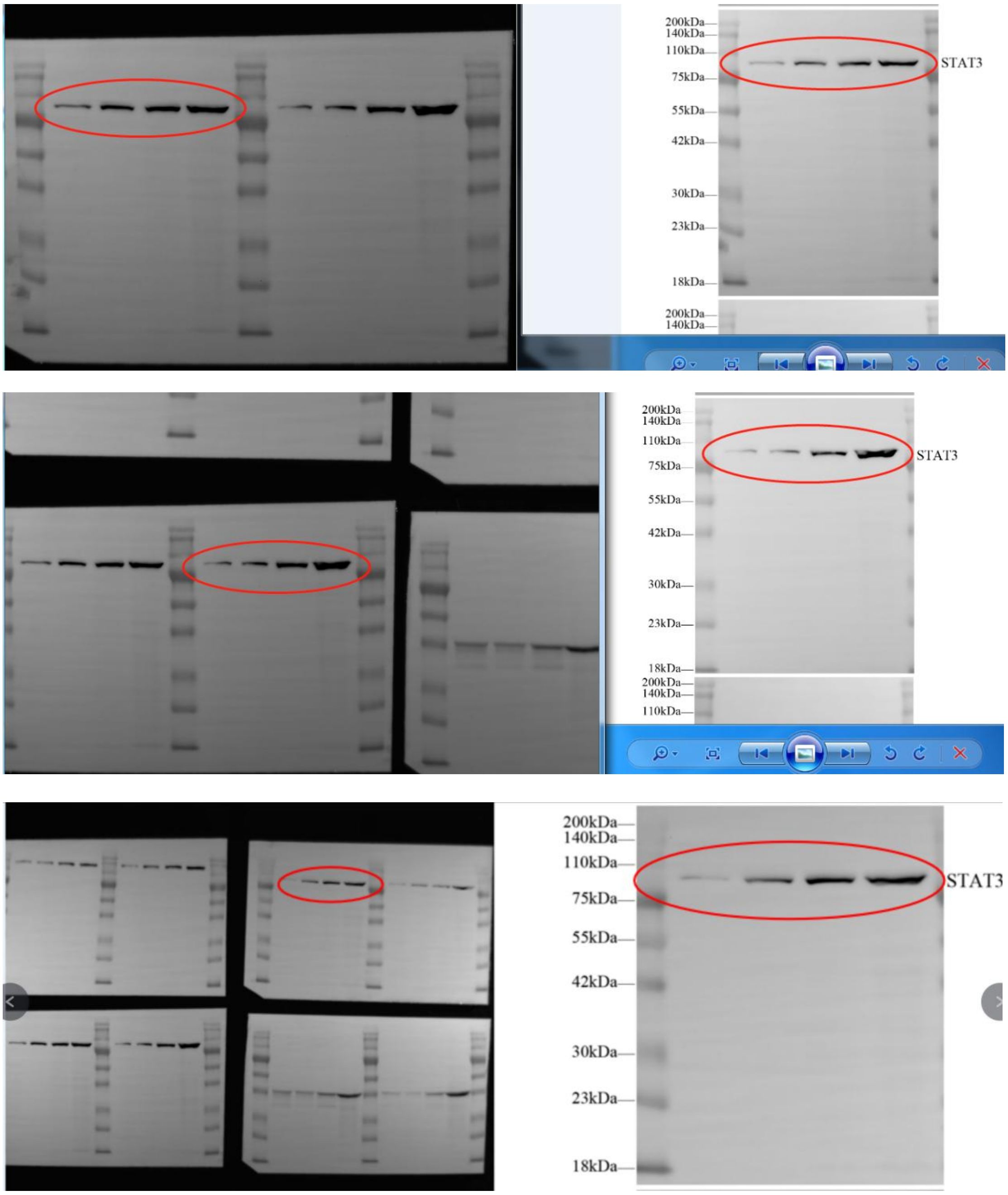

Fig. S1b: The original blots/gels of STAT3, The figure in the munuscript was cropped from the first one. The red circle on the left corresponded to the red circle on the right.

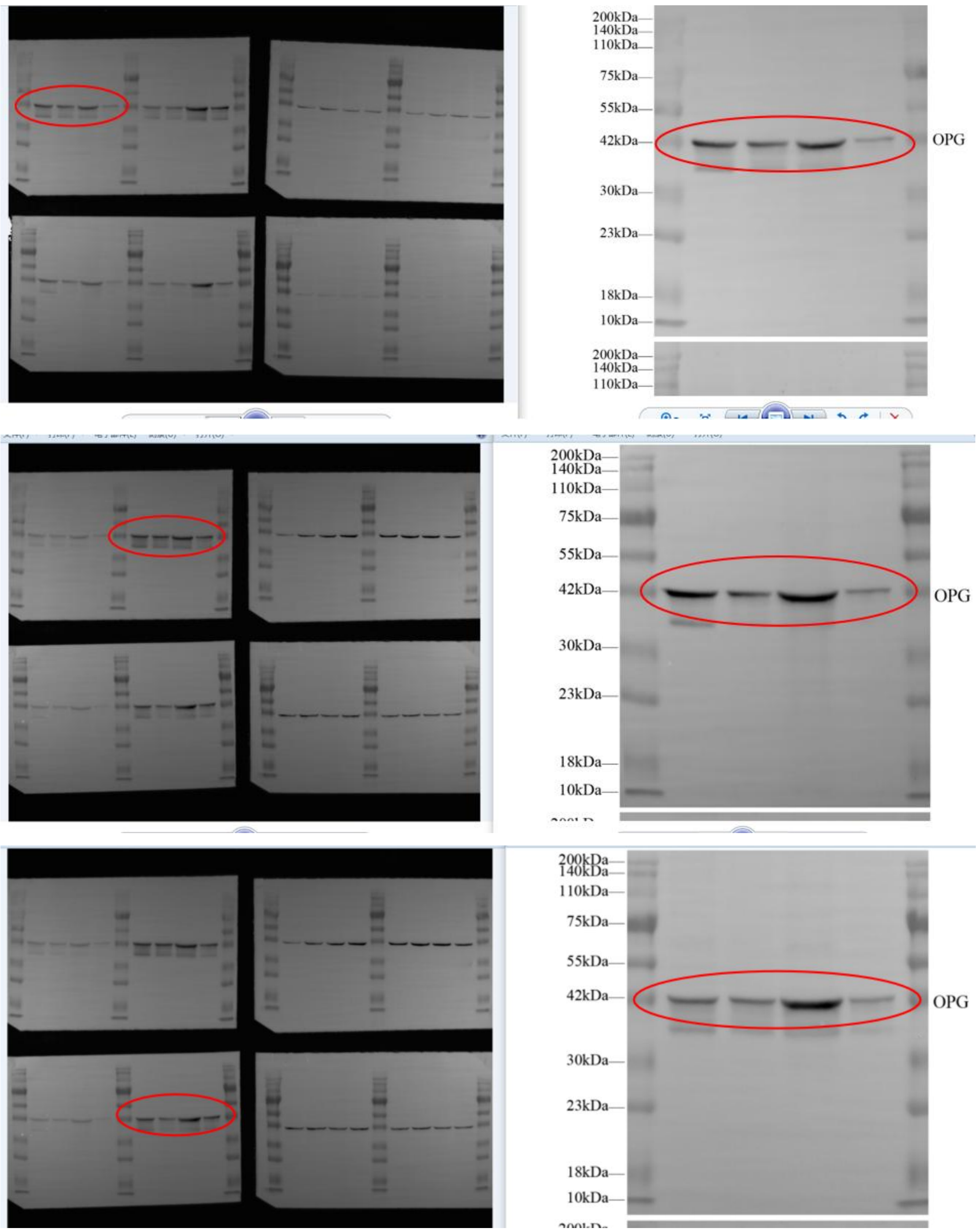

Fig. S1c: The original blots/gels of OPG, The figure in the munuscript was cropped from the first one. The red circle on the left corresponded to the red circle on the right.

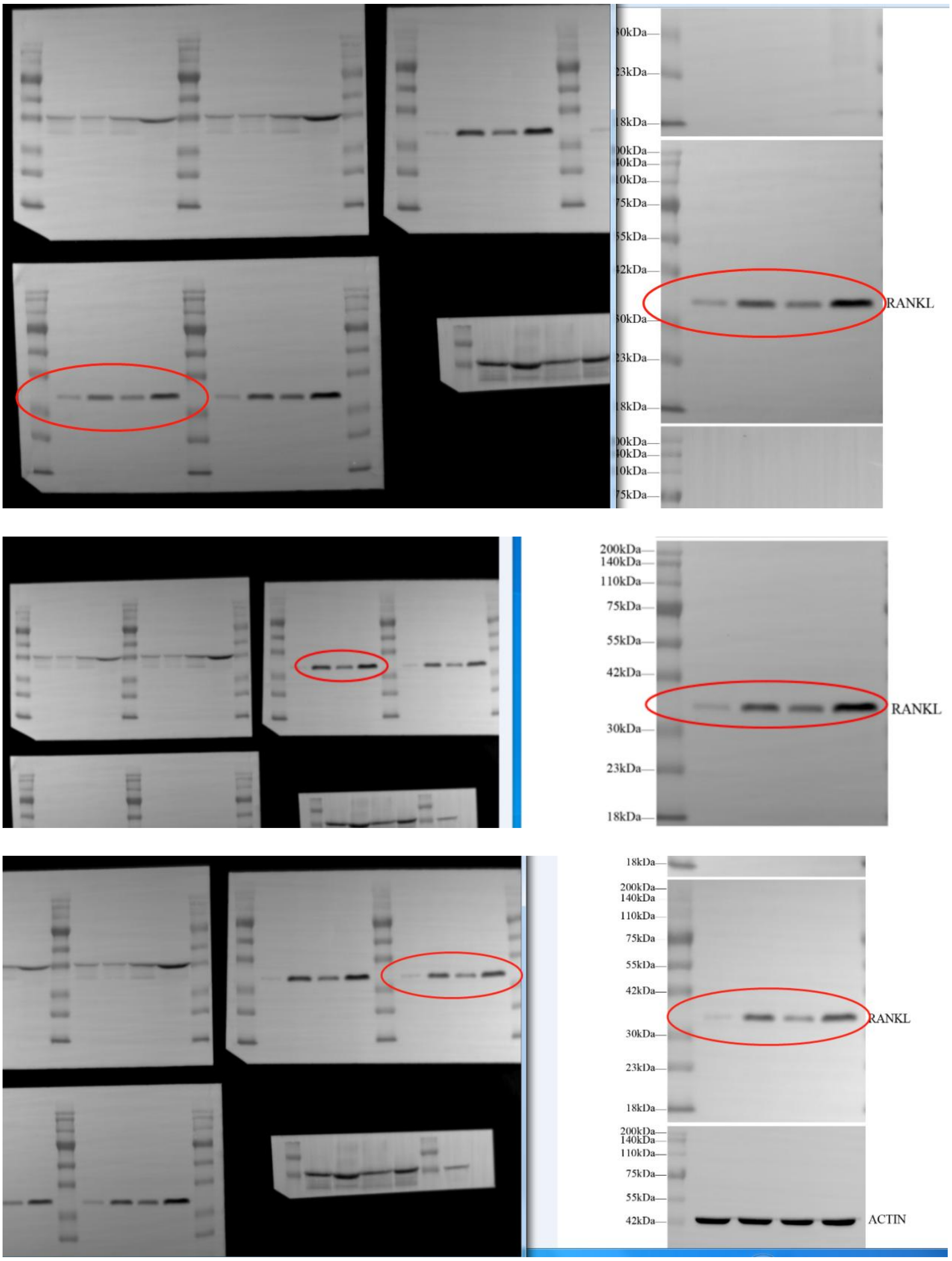

Fig. S1d: The original blots/gels of RANKL, The figure in the munuscript was cropped from the first one. The red circle on the left corresponded to the red circle on the right.

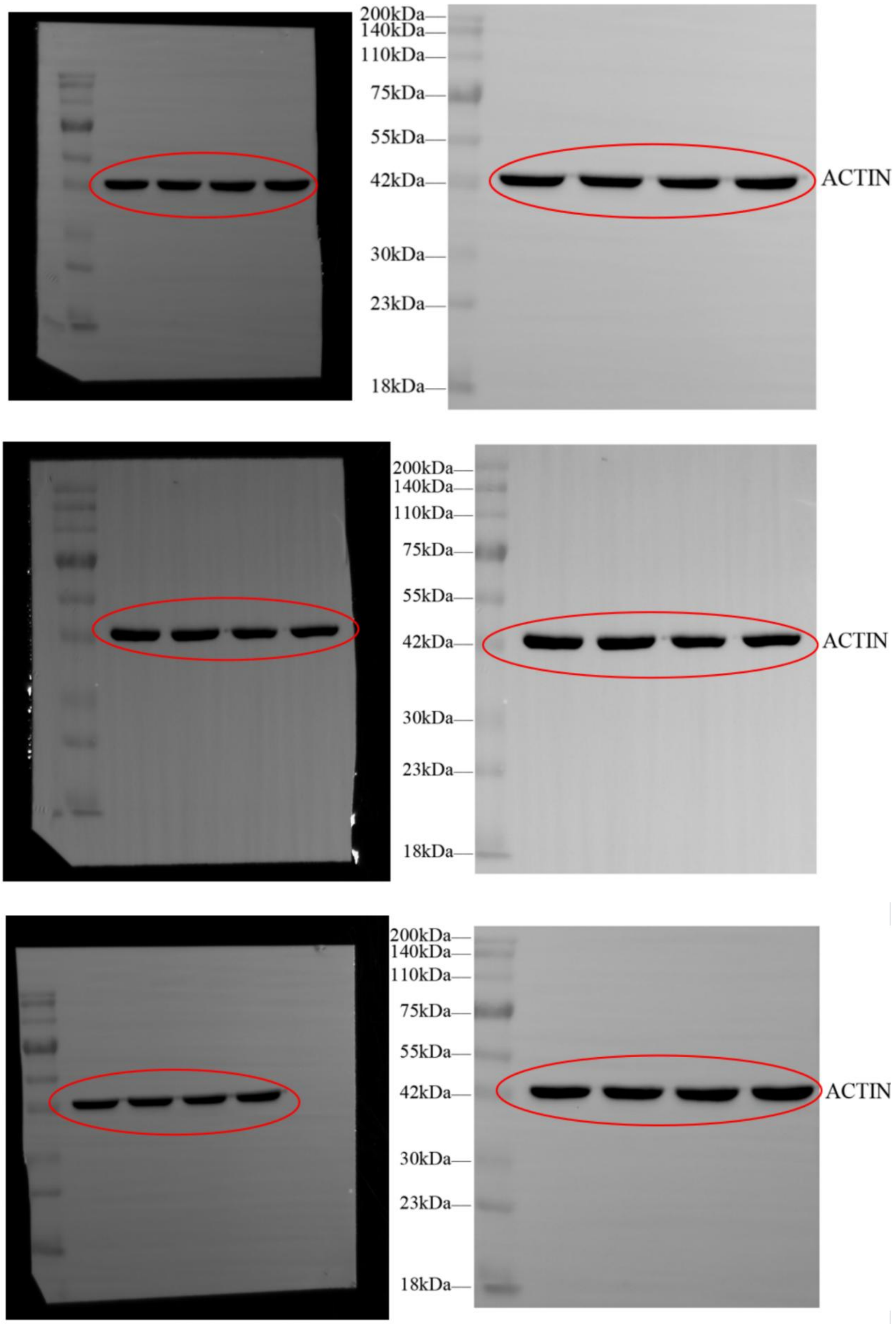

Fig. S1e: The original blots/gels of ACTIN, The figure in the munuscript was cropped from the first one. The red circle on the left corresponded

to the red circle on the right.
